# Hypothyroidism among Patients Visiting the Department of Biochemistry in Central Laboratory of a Tertiary Care Center: A Descriptive Cross-Sectional Study

**DOI:** 10.31729/jnma.8163

**Published:** 2023-05-31

**Authors:** Buddhi Raj Pokhrel, Amit Chandra Jha, Rachita Ghimire, Jharana Shrestha, Binaya Tamang, Narayan Gautam, Tapeshwar Yadav, Sakar Babu Gharti

**Affiliations:** 1 Department of Biochemistry, Universal College of Medical Sciences, Ranigaun, Rupandehi, Nepal; 2 Department of Biochemistry, National Medical College, Birguni, Parsa, Nepal; 3 Universal College of Medical Sciences, Ranigaun, Rupandehi, Nepal; 4 Department of Biochemistry, Madan Bhandari Academy of Health Sciences, TCN Road, Hetauda, Nepal; 5 Pathari Municipality Hospital, Pathari Shanishchare, Morang, Nepal

**Keywords:** *hypothyroidism*, *Nepal*, *thyroid-stimulating hormone*

## Abstract

**Introduction:**

The global burden of thyroid disorders, especially hypothyroidism, is high and increasing. Prevalence studies of such disorders are limited in Nepal. The aim of this study was to find out the prevalence of hypothyroidism among patients visiting the Department of Biochemistry in the central laboratory of a tertiary care centre.

**Methods:**

A descriptive cross-sectional study was conducted among patients visiting the Department of Biochemistry in the central laboratory from 1 August 2020 to 31 July 2021 after taking ethical approval from the Institutional Review Committee (Reference number: UCMS/IRC/054/20). Patients of all age groups and gender were considered. Hypothyroid patients were identified based on the thyroid function parameters. They were further categorized as sub-clinical and overt hypothyroid. A convenience sampling method was used. Point estimate and 95% Confidence Interval were calculated.

**Results:**

Among 3,010 patients, the prevalence of hypothyroidism was seen in 770 (25.58%) (24.02-27.14, 95% Confidence Interval). Out of total hypothyroid patients, 555 (72.08%) were females. Overt hypothyroidism 519 (67.40%) was the most prevalent hypothyroid disorder, followed by subclinical hypothyroidism 251 (32.60%).

**Conclusions:**

The prevalence of hypothyroidism among patients visiting the Department of Biochemistry in the central laboratory of a tertiary care centre was higher than in other studies done in similar settings.

## INTRODUCTION

Thyroid disorders are common and may have severe health consequences. They can be broadly classified as hyperthyroidism and hypothyroidism. Hypothyroidism is more prevalent, affecting 0.2-5.3% of populations worldwide. It is even more common in the Asian region. Both hyperthyroidism and hypothyroidism have a female preponderance, especially in pregnant females, and affect people of higher age groups.^1-3^

Several hospital-based studies from different regions of Nepal have reported the prevalence of hypothyroidism ranging from 12.4-30.4%.^4-7^ In the absence of nationwide prevalence studies, hospital-based data provide a glimpse of the overall pattern. More region-wise studies will help to assess the disease burden and are relevant.

The aim of this study was to find out the prevalence of hypothyroidism among patients visiting the Department of Biochemistry in the central laboratory of a tertiary care centre.

## METHODS

A descriptive cross-sectional study was conducted at the Department of Biochemistry of Universal College of Medical Sciences and Teaching Hospital Ranigaun, Rupandehi, Nepal from 1 August 2020 to 31 July 2021. The ethical approval was taken from the Institutional Review Committee of UCMSTH (Reference number: UCMS/IRC/054/20). Patients of all age groups and gender who were sent for the evaluation of thyroid function test (TFT) at the laboratory were included. Those with incomplete TFT parameters were excluded. A convenience sampling method was used. The sample size was calculated using the following formula:


$$\begin{array}{ll} \text{n} & ={\text{Z}}^{2}\times \frac{\text{p}\times \text{q}}{{\text{e}}^{\text{2}}}\\ & =1.96^{2}\times \frac{0.50\times 0.50}{0.02^{2}}\\ & =2401 \end{array}$$

Where,

n = minimum required sample size
Z= 1.96 at 95% Confidence Interval (CI)
p = prevalence taken as 50% for maximum sample size calculation
q = 1-p
e = margin of error, 2%

The minimum required sample size was 2401. After adding 10% non response rate, the calculated sample size was 2641. However, a sample size of 3010 was taken. The data from all the patients who fulfilled the above criteria within the study duration and provided consent were evaluated. Consent for children and adolescents was obtained from their parents. The patients were informed in detail about the study and were ensured of the confidentiality of the data. Both verbal and written consent was obtained from the participants. All the participants were required to fill out a study proforma that included their socio-demographic parameters. Their TFT status was added to the proforma after evaluation. The TFT parameters were evaluated on the serum samples using a chemiluminescence assay (Maglumi 2000). The following reference ranges were considered, as per the manufacturer's instructions; fT_3_: 2.0-4.2 pg/ml, fT_4_: 8.9-17.2 pg/ml, and TSH: 0.3-4.5 μIU/ml. The following criteria were used to categorize the participants: Euthyroid: All TFT parameters within the reference range; Overt hypothyroid: primary hypothyroid disorders with increased TSH levels and decreased fT_4_ levels; Sub-clinical hypothyroid: primary hypothyroid disorders with increased TSH levels but normal fT_4_ levels.^2,8^

Data was entered in Microsoft Excel 2016 and analysis was done using IBM Statistics SPSS 16.0. Point estimate and 95% CI were calculated.

## RESULTS

**Figure 1:**
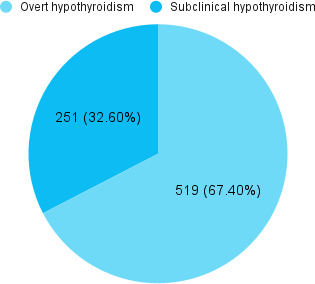
Distribution of hypothyroidism (n= 770)

Among 3,010 patients, the prevalence of hypothyroidism was seen in 770 (25.58%) (24.02-27.14, 95% CI). Overt hypothyroidism was seen among 519 (17.24%) and sub-clinical hypothyroidism was seen among 251 (8.34%) participants (Figure 1).

The median age of the participants was 37 (26-51) years. Among 770 hypothyroid patients, 555 (72.08%) were females (Table 1).

**Table 1:** Gender and age-wise distribution (n= 770).

		Overt n (%)	Sub-clinical n (%)
**Gender**	Male	151 (19.61)	64 (8.31)
	Female	368 (47.79)	187 (24.29)
**Age group**	<15	38 (4.94)	12 (1.56)
	16-30	138 (17.92)	73 (9.48)
	31-50	192 (24.94)	94 (12.21)
	>50	151 (19.61)	72 (9.35)

The median fT_3_, fT_4_, and TSH level of the male was 1.88 (1.66-2.13), 8.10 (7.33-10.11), and 7.58 (5.81-12.09) respectively (Table 2).

Pokhrel et al. Hypothyroidism among Patients Visiting the Department of Biochemistry in Central Laboratory of a Tertiary Care Center...

**Table 2:** Gender and age-wise distribution of TFT parameters (n= 770).

Variables	fT3 (pg/ml) Median (IQR)	fT4 (pg/ml) Median (IQR)	TSH (plU/ml) Median (IQR)
**Gender**			
Male	1.88 (1.66-2.13)	8.10 (7.33-10.11)	7.58 (5.81-12.09)
Female	1.89 (2.37-3.52)	8.12 (7.32-10.51)	7.15 (5.67-13.05)
**Age-group**			
≤15	1.88 (1.57-2.16)	7.83 (7.10-8.54)	8.20 (5.81-16.34)
16-30	1.91 (1.65-2.23)	8.14 (7.33-10.60)	6.94 (5.57-13.54)
31-50	1.89 (1.65-2.20)	8.11 (7.38-10.39)	6.92 (5.62-11.43)
>50	1.87 (1.66-2.16)	8.09 (7.32-10.33)	7.61 (6.00-13.46)

## DISCUSSION

The overall prevalence of hypothyroid disorders was 25.58%. The most common hypothyroid disorder was overt hypothyroidism (17.2%) followed by sub-clinical hypothyroidism (8.3%). None of the patients were identified as central hypothyroid. All the disorders had female preponderance.

Findings from other studies from Nepal are slightly variable. The differences in the prevalence (12.4-30.4%) may be attributed to one of the following factors: geographic variation, the difference in methods of estimation and the kits used, and the use of different reference ranges for the diagnosis.^4-7,9,10^ The prevalence of sub-clinical hypothyroidism also varied widely in some of these studies (11.4% - 24.8%) and was either comparable to overt hypothyroidism or much greater.^6,10^ In contrast, our study showed a higher prevalence of overt hypothyroidism than sub-clinical hypothyroidism. The lower prevalence of sub-clinical hypothyroidism compared to overt hypothyroidism in our study may be due to the obscure clinical picture that might have led to the missed TFT evaluation.

The global scenario is not much different, although the prevalence range is quite varied across regions. A review from India reported the prevalence of overt hypothyroidism and sub-clinical hypothyroidism to be 3.9% and 9.4%, respectively.^11^ A study from Japan reported a lower prevalence of hypothyroidism (6.5%) among adults.^12^ Much further, a longitudinal study from Brazil reported the prevalence of hypothyroidism at 7.4%.^13^ A meta-analysis from Europe revealed an even lower prevalence of thyroid dysfunctions (3.82%), where 3.05% were hypothyroid.^14^ A large-scale National Health and Nutrition Examination Survey (NHANES III) reported that 5.9% population of the United States had thyroid dysfunction, where 4.6% were hypothyroid.^15^

The reasons for gender disparity in the prevalence of thyroid disorders are not completely understood. Many thyroid disorders are autoimmune in nature. The autoimmune disorders are generally more prevalent in females, probably secondary to the effects of sex steroid hormones in the immune system.^16,17^

There are a few limitations to our study that needs to be mentioned. Being a hospital-based study, our findings may not represent the accurate prevalence of hypothyroidism in the western population of Nepal. Specifically, the sub-clinical hypothyroidism might have been underestimated in our study as only the suspected patients were sent for laboratory analysis. Further large-scale community-based screenings of hypothyroidism are needed.

## CONCLUSIONS

The prevalence of hypothyroidism among patients visiting the Department of Biochemistry in the central laboratory of a tertiary care centre is higher than other studies done in similar settings with a higher prevalence of overt hypothyroidism, followed by sub-clinical hypothyroidism.
